# Evaluating histone H3.1 as a biomarker for acute ischemic stroke: insights into NETs and stroke pathophysiology

**DOI:** 10.1007/s44313-024-00047-1

**Published:** 2024-12-03

**Authors:** Suji Park, Jae-Ryong Shim, Ri-Young Goh, Dae-Hyun Kim, Jin-Yeong Han

**Affiliations:** 1 Department of Laboratory Medicine, Dong-A University College of Medicine, Busan, Korea; 2 Department of Neurology, Dong-A University College of Medicine, Busan, Korea

**Keywords:** Neutrophil extracellular traps, Ischemic stroke, Histone, Biomarker, Immunothrombosis

## Abstract

The diagnosis of acute ischemic stroke (AIS) can be challenging when neuroimaging findings are normal or equivocal. Neutrophil extracellular traps (NETs), particularly histone H3.1, have potential as biomarkers for AIS. This study evaluated NETs, specifically histone H3.1, as diagnostic biomarkers for AIS. This prospective study included 89 patients with AIS and 20 healthy controls. Plasma histone H3.1 levels were measured using the Nu.Q® H3.1 enzyme-linked immunosorbent assay (ELISA). Seven cytokines were analyzed using a bead-based immunoassay. Statistical analyses were used to compare histone H3.1 levels between groups and evaluate correlations with clinical parameters and cytokines. Histone H3.1 levels were significantly higher in patients with AIS (271.05 ± 33.40 ng/mL) versus controls (95.33 ± 12.86 ng/mL, *p* < 0.001). Multivariable logistic regression identified H3.1 as an independent risk factor for AIS (*p* = 0.006), with an area under the curve of 0.907. Significant correlations were found between H3.1, interleukin-6 (0.290, *p* = 0.013) and vascular cell adhesion molecule 1 (0.297, *p* = 0.011). In conclusion, the NETs H3.1 ELISA test is a reliable new diagnostic option that supports the diagnosis of AIS.

According to the Global Burden of Diseases, Injuries, and Risk Factor Study, stroke is the second leading cause of death globally and results from the occlusion of cerebral blood flow due to intravascular thrombus formation [1]. Given the narrow therapeutic window of only a few hours, rapid assessment of acute stroke victims is critical for determining their eligibility for thrombolytic therapy. Currently, stroke diagnosis relies on clinical examination supplemented by neuroimaging techniques. While primary care physicians with extensive exposure to stroke cases have shown a diagnostic accuracy of 92%, the reliability decreases with less experienced practitioners. Initial neurological imaging primarily aims to differentiate between hemorrhagic and ischemic stroke and promptly exclude stroke mimics. In this context, blood biomarkers for diagnosing stroke, differentiating stroke types, and predicting initial or recurring stroke are extremely valuable, especially in challenging cases with normal or equivocal neuroimaging findings [2]. Among the several potential biomarkers, neutrophil extracellular traps (NETs) are promising owing to their involvement in the pathophysiology of ischemic stroke. Aberrant thrombus formation involves abnormal blood coagulation, platelet activation, and the participation of monocytes and neutrophils, which are components of the innate immune system [3, 4]. This interaction, termed immunothrombosis, culminates in the formation of NETs, which are web-like structures released by activated neutrophils composed of decondensed chromatin fibers and antimicrobial granular proteins such as histones, myeloperoxidase (MPO), neutrophil elastase (NE), and cathepsin G [5, 6]. Previous studies have confirmed the presence of NETs in the brains of patients with ischemic stroke and their correlation with stroke outcomes [5]. However, the complexity of brain tissue types and incomplete knowledge of cerebral physiology have contributed to the current lack of stroke-specific biomarkers. Additionally, the lack of standardized methods for NETs poses a challenge. Histone H3.1 has been implicated in the formation of NETs; consequently, the quantification of circulating H3.1-nucleosomes has emerged as a reliable surrogate marker for assessing NET levels in the plasma [7]. Additionally, the Nu.Q® H3.1 enzyme-linked immunosorbent assay (ELISA) offers an effective and standardized approach for quantifying NET formation in blood samples. Therefore, this study aimed to utilize H3.1 for measuring NETs, and to evaluate whether they could serve as novel markers in patients with AIS.

Between January and June 2023, we prospectively enrolled 89 consecutive patients newly diagnosed with AIS at a single institution. The diagnosis was based on patient symptoms, physical examination, Doppler ultrasonography, brain magnetic resonance imaging, and computed tomography findings. For the control group, we selected 20 adults undergoing routine health screening with normal complete blood count (CBC) results. Clinical and laboratory data were obtained from the medical records. This study was approved by the local ethics committee (approval number: DAUHIRB-23–195). K2 ethylenediaminetetraacetic acid plasma samples were stored at –80 °C, and before analysis, thawed in a 37°C water bath for up to 10 min, and gently mixed. All the tests were completed within 4h of thawing. Histone H3.1 were measured using the Nu.Q H3.1 ELISA assay (Belgian Volition SRL., Isnes, Belgium), which quantifies histone within intact nucleosome using specific anti-histone and anti-nucleosome antibodies. Following the manufacturer's guidelines, any sample with a coefficient of variation (CV) above 20% was reassessed. In this study, all the samples were measured in duplicate, with none exceeding 20% CV. The detection range of the assay was 22.7–650 ng/mL. We measured seven cytokines (interferon [IFN]-γ, interleukin [IL]-β, IL-18, IL-6, P-selectin, tumor necrosis factor [TNF]-α, and vascular cell adhesion molecule [VCAM]−1) using a bead-based immunoassay with a multi-cytokine detection kit (R&D Systems, Inc., Minneapolis, MN, USA), following the manufacturer's instructions. Statistical analyses were conducted using SPSS version 29.0.2.0 (IBM Corp., Armonk, NY, USA), with a significance threshold of *p* < 0.05. Data are expressed as numbers with percentages or mean ± standard deviation. Continuous variables were compared using the Mann–Whitney U test, whereas categorical variables were analyzed using the chi-square test. Distributions of histone H3.1 levels in the AIS and control groups were visualized using box and whisker plots (Fig. 1). Multivariable logistic regression analyses were performed on variables with *p* < 0.05 in univariable analyses, using both the enter and forward likelihood ratio test methods. Receiver operating characteristic (ROC) curves were used to calculate the area under the curve (AUC) for H3.1. Correlations between nucleosomes and inflammatory cytokines were assessed using Spearman correlation coefficients. The baseline variables analyzed in the AIS and control groups included age, sex, underlying diseases (diabetes mellitus [DM], hypertension, and dyslipidemia), social history (alcohol intake and current smoking), and laboratory data (white blood cell [WBC] and platelet counts). Significant differences were found between the AIS and control groups in terms of age, history of DM, hypertension, current smoking status, and WBC counts. The baseline characteristics of the study population are summarized in Table 1. Circulating NET levels, measured by H3.1 concentrations, were significantly higher in the AIS group than in the control group (AIS: 271.05 ± 33.40 ng/mL; control: 95.33 ± 12.86 ng/mL, *p* < 0.001). Logistic regression analyses determined H3.1 cutoff values using Youden’s index. Univariable analyses revealed that hypertension, WBC count, and H3.1 levels were significantly associated with AIS (*p* = 0.008, *p* < 0.001, and *p* = 0.006, respectively). Multivariable analysis identified H3.1 concentration as a significant risk factor for AIS (*p* = 0.006; odds ratio: 0.082; 95% confidence interval: 0.014–0.492). Table 2 presents the results of these analyses. ROC curve analysis for AIS risk factors yielded an AUC of 0.907 for H3.1 concentration with an optimal cutoff of 125.81 ng/mL (sensitivity: 0.70; specificity: 0.85; Fig. 2).

**Fig. 1:**
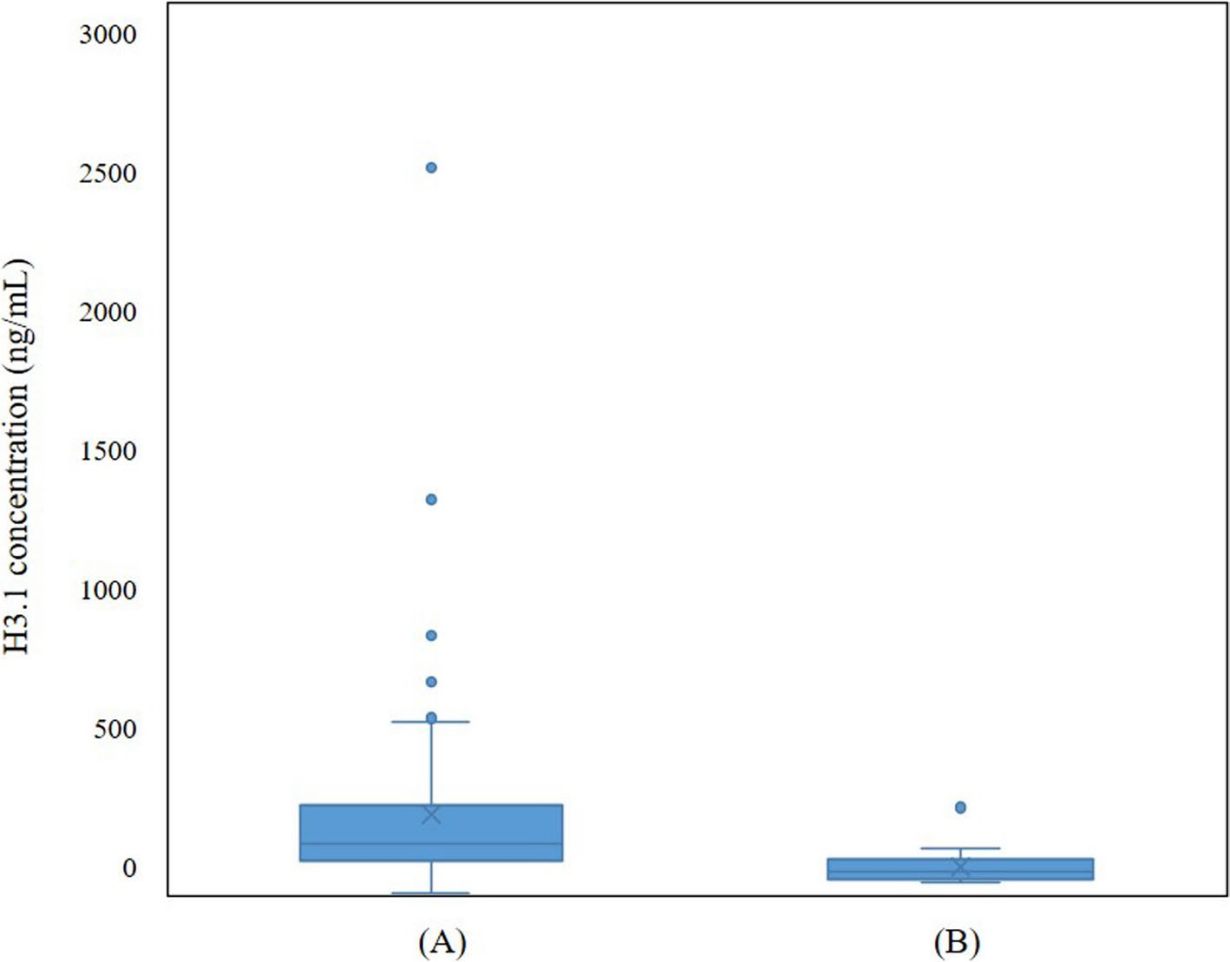
Box and whisker plots of distributions of histone H3.1 levels in the (A) acute ischemic stroke group and (B) control group

**Table 1:** Baseline characteristics of the study population

Variables	AIS (*N* = 89)	Control (*N* = 20)	*P* (AIS vs. control)
Demographic characteristics			
Age (y), mean ± SD	70.8 ± 11.5	66.1 ± 4.6	0.015
Male, no. (%)	55 (62.0)	10 (50.0)	0.331
Underlying diseases			
Diabetes mellitus, no. (%)	34 (38.0)	3 (15.0)	0.048
Hypertension no. (%)	57 (64.0)	6 (30.0)	0.005
Dyslipidemia, no. (%)	16 (18.0)	6 (30.0)	0.226
Social History			
Alcohol intake, no. (%)	26 (29.0)	7 (35.0)	0.661
Current smoking, no. (%)	23 (26.0)	1 (5.0)	0.042
Laboratory data			
WBC (× 10^9^/L)	8.69 ± 3.1	5.9 ± 1.0	< 0.001
Platelet (× 10^9^/L)	236.5 ± 44.5	246.5 ± 44.56	0.232

*AIS* acute ischemic stroke, *SD* standard deviation, *WBC* white blood cells

**Table 2:** Results of the logistic regression analyses for H3.1 concentration and other variable in AIS patients

Variable	Univariate			Multivariate		
	**OR**	**95% CI**	***P***	**OR**	**95% CI**	***P***
Hypertension						
	0.241	0.084–0.687	**0.008**	0.246	0.055–1.103	0.067
WBC count						
	0.999	0.999–1.000	** < 0.001**	0.999	0.999–1.000	**0.012**
H3.1						
	0.077	0.021–0.284	**0.006**	0.082	0.014–0.492	**0.006**

*OR* odd ratio, *CI* confidence interval

**Fig. 2:**
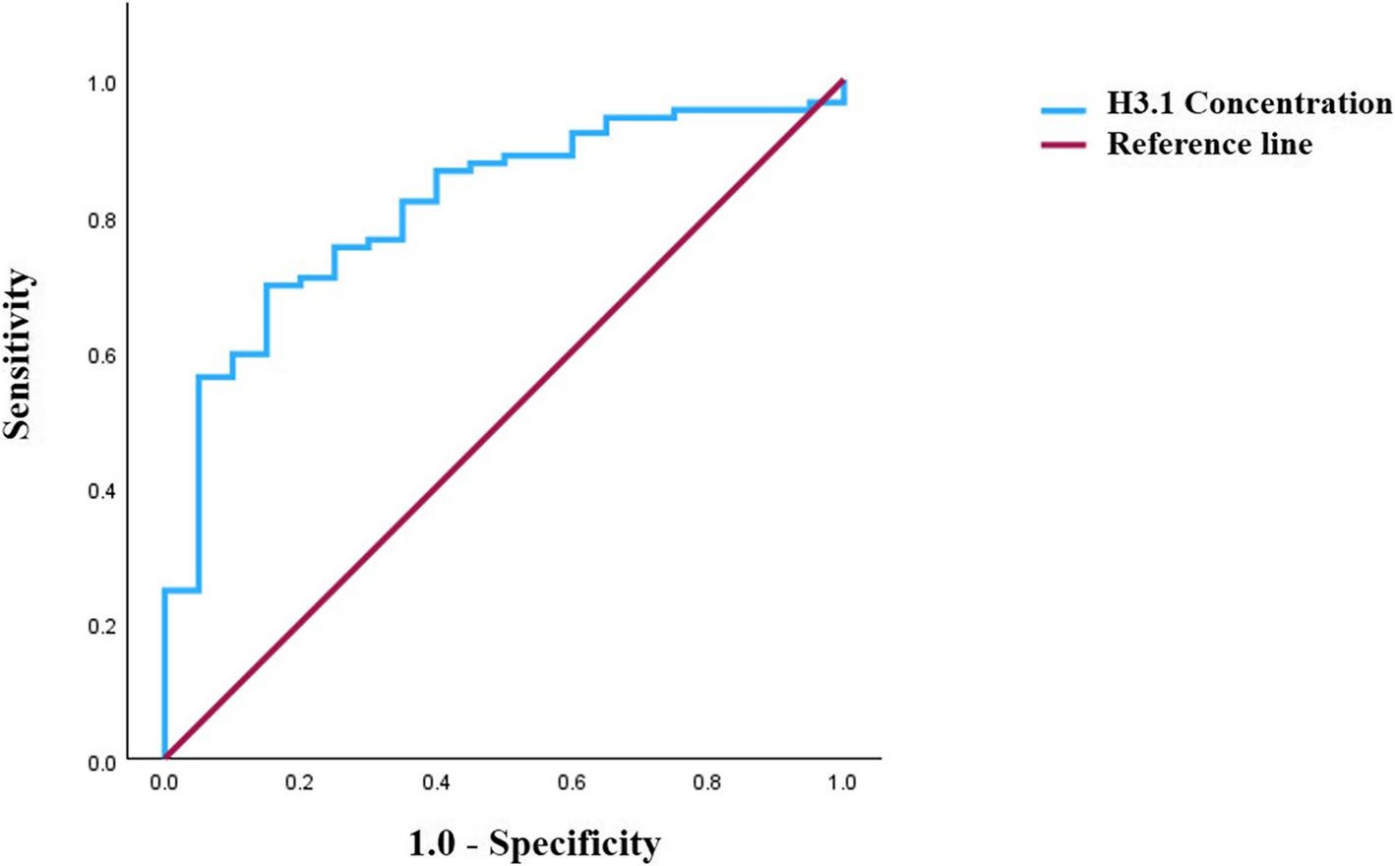
Receiver operating characteristic (ROC) curves for H3.1 concentration in acute ischemic stroke patients

The interplay between thrombosis and inflammation (immunothrombosis) is a key driver of stroke. We assessed the association of NETs with five pro-inflammatory cytokines (IFN-γ, IL-1β, IL-6, IL-18, and TNF-α) and two cytokines they include (P-selectin, VCAM-1), using a multi-cytokine detection kit. Among the 62 patients with sufficient sample volumes (> 75 uL), IL-6 and VCAM-1 showed significant Spearman correlation coefficients with H3.1 (0.290 and 0.297, respectively).

This study evaluated whether H3.1, a NET marker, could aid in the diagnosis of AIS. The role of NETs as biomarkers of AIS is debated because of their involvement in inflammation and thrombosis, which are critical in stroke pathophysiology. NETs can be detected shortly after stroke onset, potentially enabling earlier diagnosis and intervention, which is crucial given the narrow therapeutic window for stroke treatment [8]. The investigation of NETs can provide insights into stroke mechanisms, particularly the interplay between inflammation and thrombosis, and potentially identify new therapeutic targets. Elevated NET levels correlate with stroke severity and outcomes, suggesting their potential for prognosis and treatment guidance [9]. However, NET levels vary significantly among individuals over time, which complicates the establishment of reliable diagnostic thresholds [8]. Elevated NET levels are not specific to stroke and can result in false positives, complicating the differential diagnosis. The detection and quantification of NETs require specialized techniques and equipment, thereby limiting their widespread use [9].

In this study, patients with AIS exhibited significantly higher levels of H3.1 compared to controls. H3.1 emerged as a significant risk factor for AIS in the multivariable analysis (*p* = 0.006). ROC curve analysis showed an AUC of 0.907 for H3.1, indicating strong diagnostic potential. Correlations were found between H3.1 and the cytokines IL-6 and VCAM-1, suggesting that combining these markers could enhance diagnostic specificity.

Previous studies have supported the potential of NETs as biomarkers when neuroimaging findings are normal or equivocal. Plasma DNA levels were found to be elevated threefold in patients with AIS, correlating positively with infarct size. Subsequent studies have confirmed that NETs are associated with thrombosis, brain injury, and poor prognosis [9–11]. NETs have been detected in the brain tissue of patients with ischemic stroke, as evidenced by neutrophil-specific markers, such as NE, MPO, and citrullinated histones [5]. Recent data from prospective studies have shown significant formation of NETs in the thrombi of patients with AIS undergoing mechanical thrombectomy and in the plasma of patients with stroke [12]. In a mouse study, intravascular brain NETs infiltrated the brain within 24 h after subarachnoid hemorrhage (SAH), participated in delayed cerebral ischemia (DCI), and were identified in the blood [13]. In a concurrent prospective study, it was confirmed that the formation of NETs was suppressed and decomposed in an SAH mouse model, thereby improving cerebral blood flow and reducing neurological deficits and DCI. An increase in NETs has also been observed in patients who continuously developed DCI after SAH, confirming that NETs could be used as potential therapeutic targets and acute-phase markers [14].

This study has some limitations, including a small sample size, single-time measurement, and a single-center design, which may limit the generalizability of our findings. It also did not consider the temporal changes in NET levels after stroke onset, which could have affected the interpretation. However, since current stroke diagnoses rely on imaging studies and clinical symptoms can be ambiguous, NETs could serve as supplementary diagnostic tools. They can be measured using blood tests, providing valuable information even in resource-limited areas without access to skilled neurologists or radiologists. This can help determine the need for further diagnosis and treatment, offering a time-efficient approach.

In conclusion, this study provides evidence that NETs, particularly histone H3.1, are significantly elevated in patients with AIS, highlighting their potential as biomarkers for stroke. However, further research involving larger cohorts is necessary to validate our results and fully establish their clinical utility.

## Data Availability

No datasets were generated or analysed during the current study.

## References

[1] Lozano R, et al. Global and regional mortality from 235 causes of death for 20 age groups in 1990 and 2010: A systematic analysis for the Global Burden of Disease Study 2010. Lancet. 2012;380(9859):2095–128. 10.1016/S0140-6736(12)61728-0. 10.1016/S0140-6736(12)61728-0 PMC10790329 23245604

[2] Saenger AK, Christenson RH. Stroke biomarkers: Progress and challenges for diagnosis, prognosis, differentiation, and treatment. Clin Chem. 2010;56(1):21–33. 10.1373/clinchem.2009.133801. 19926776 10.1373/clinchem.2009.133801

[3] Stark K, Massberg S. Interplay between inflammation and thrombosis in cardiovascular pathology. Nat Rev Cardiol. 2021;18(9):666–82. 10.1038/s41569-021-00552-1. 33958774 10.1038/s41569-021-00552-1 PMC8100938

[4] Alsharidah AS. Diabetes mellitus and diabetic nephropathy : a review of the literature on hemostatic changes in coagulation and thrombosis. Blood Res. 2022;57(2):101–5. 35620906 10.5045/br.2022.2021204 PMC9242838

[5] Denorme F, et al. Neutrophil extracellular traps regulate ischemic stroke brain injury. J Clin Invest. 2022;132(10):e154225. 10.1172/JCI154225. 35358095 10.1172/JCI154225 PMC9106355

[6] Islam MM, Takeyama N. Role of Neutrophil Extracellular Traps in Health and Disease Pathophysiology: Recent Insights and Advances. Int J Mol Sci. 2023;24(21):15805. 10.3390/ijms242115805. 37958788 10.3390/ijms242115805 PMC10649138

[7] Rahimi MH, Bidar F, Lukaszewicz AC, Garnier L, Gay LP. Association of pronounced elevation of NET formation and nucleosome biomarkers with mortality in patients with septic shock. Ann Intensive Care. 2023. 10.1186/s13613-023-01204-y. 10.1186/s13613-023-01204-y PMC10581968 37847336

[8] Supanc V, Biloglav Z, Kes V, Demarin V. Role of cell adhesion molecules in acute ischemic stroke. Ann Saudi Med. 2011;31(4):365–70. 10.4103/0256-4947.83217. 21808112 10.4103/0256-4947.83217 PMC3156512

[9] O’Connell GC, et al. Circulating extracellular DNA levels are acutely elevated in ischaemic stroke and associated with innate immune system activation. Brain Inj. 2017;31(10):1369–75. 10.1080/02699052.2017.1312018. 28585898 10.1080/02699052.2017.1312018

[10] Gao X, et al. Neutrophil extracellular traps mediated by platelet microvesicles promote thrombosis and brain injury in acute ischemic stroke. Cell Commun Signal. 2024;22(1):1–12. 10.1186/s12964-023-01379-8. 38233928 10.1186/s12964-023-01379-8 PMC10795390

[11] Zdanyte M, Borst O, Münzer P. NET-(works) in arterial and venous thrombo-occlusive diseases. Front Cardiovasc Med. 2023;10(May):1–12. 10.3389/fcvm.2023.1155512. 10.3389/fcvm.2023.1155512 PMC10239889 37283578

[12] Liaptsi E, et al. Targeting Neutrophil Extracellular Traps for Stroke Prognosis: A Promising Path. Neurol Int. 2023;15(4):1212–26. 10.3390/neurolint15040076. 37873833 10.3390/neurolint15040076 PMC10594510

[13] Zeineddine HA, et al. Neutrophils and Neutrophil Extracellular Traps Cause Vascular Occlusion and Delayed Cerebral Ischemia after Subarachnoid Hemorrhage in Mice. Arterioscler Thromb Vasc Biol. 2024;44(3):635–52. 10.1161/ATVBAHA.123.320224. 38299355 10.1161/ATVBAHA.123.320224 PMC10923061

[14] Mengozzi L, et al. Neutrophil Extracellular Traps and Thrombolysis Resistance: New Insights for Targeting Therapies. Stroke. 2024;55(4):963–71. 10.1161/STROKEAHA.123.045225. 38465650 10.1161/STROKEAHA.123.045225 PMC10962437

